# The effect of multidisciplinary team care on cancer management

**DOI:** 10.4314/pamj.v9i1.71195

**Published:** 2011-06-17

**Authors:** Ganiy Opeyemi Abdulrahman

**Affiliations:** 1 Cardiff University of Wales College of Medicine, School of Medicine Registry, Cardiff University, United Kingdom

**Keywords:** Multidisciplinary, cancer, team, effectiveness, malignancy, MDT

## Abstract

Over the past 15 years, the multidisciplinary team management of many medical conditions especially cancers has increasingly taken a prominent role in patient management in many hospitals and medical centres in the developed countries. In the United Kingdom, it began to gain prominence following the Calman-Heine report in 1995 which suggested that each Cancer Unit in a hospital should have in place arrangements for non-surgical oncological input into services, with a role for a non-surgical oncologist. The report further suggested that a lead clinician with a well established interest in cancer care should be appointed to organise and coordinate the whole range of cancer services provided within the Cancer Unit. Many people have argued that the multidisciplinary team management of patients has resulted in better care and improved survival. However, there are barriers to the optimal effectiveness of the multidisciplinary team. This paper aims to review various studies on the effectiveness of the multidisciplinary team in the management of cancer patients and also discuss some of the barriers to the multidisciplinary team.

## Introduction

A multidisciplinary team (MDT) is a group of health care members in different disciplines, each providing specific services to the patient with the aim of ensuring that the patient receives optimum care and support. The importance of a multidisciplinary team was particularly highlighted by the 1995 Calman-Heine report which suggests in its recommendation that each Cancer Unit in a hospital should have in place arrangements for non-surgical oncological input into services, with a role for a non-surgical oncologist and that a lead clinician with a well established interest in cancer care should be appointed to organise and coordinate the whole range of cancer services provided within the Cancer Unit [1]. The report went further to recommend that other services such as physiotherapy, nursing, dietetics, speech therapy, chaplaincy and social services should all be easily available [1]. Ever since this report was published, multidisciplinary team for cancer patients is now well established in many hospitals across England and Wales [2–7]. This paper is aimed at discussing the effectiveness of multidisciplinary team management of cancer patients.

### The need for multidisciplinary care

Previous management of cancer patients before the idea of MDT management was conceived involved the referral of patients from one clinician to another at various stages of diagnosis and treatment without an integrated approach, which can be an overwhelming and confusing experience for a patient. This resulted in uncoordinated patient care and low patient satisfaction with services.

### South East Wales

The National Health Service Wales (NHS Wales) states that a typical MDT should include [8]: a lead clinician, a team co-ordinator/secretary, a surgeon, an oncologist, a radiologist, a histo-pathologist, a clinical nurse specialist and specialist palliative care nurse.

## Methods

Thorough search of the English literature was conducted using search engines such as PubMed, Google and Medline with words that include: “multidisciplinary team”, “effectiveness of MDM” and “multidisciplinary team management of cancer”. Studies that evaluated team working effectiveness in the management of patients with cancer were included. Specific data such as number of patients involved in the studies, methodology of the studies and the outcome of the studies were noted.

## Results

A number of studies have been carried out to evaluate the effectiveness of MDT management of cancer patients. In a cohort study in the United States, 269 patients who were diagnosed with urological cancer outside a particular large health institution and then presented to the large health institution for diagnostic or treatment consideration were studied [9]. The cohort included patients with the diagnosis of cancer of the prostate (34%), bladder (23%), kidney (35%) and testicle (5%). The researchers state that all the cases were reviewed and discussed at a cancer board in the presence of all members of the different subspecialties. Following the MDT meeting, changes in diagnosis were made in 23% of the bladder cases and 17% of renal cases. Also, changes in treatment were made in 44% of the bladder cancer cases, 36% of renal cases and 29% of testicular cancer cases [9]. This result suggests that a multidisciplinary team approach affects the diagnostic and management decisions in a considerable number of patients with a newly diagnosed urologic malignancy.

In another study aimed at evaluating the effect of a colorectal multidisciplinary team in the United Kingdom, 310 patients were studied following the establishment of a multidisciplinary cancer team in a hospital [10]. The pre-MDT cohort over a 5-year period prior to the establishment of a MDT was 176 and the post-MDT cohort was 134 [10]. The study shows that a significant number of patients were prescribed adjuvant chemotherapy in the post-MDT cohort and three-year survival for Duke C patients was 58% in the pre-MDT group, compared to 66% in the post –MDT group [10]. This study suggests that the establishment of an MDT has resulted in a significant increase in the number of patients undergoing adjuvant chemotherapy which resulted in an increase in the 3-year survival of Duke C patients.

In a hospital in Glasgow, 243 patients with inoperable non-small-cell lung cancer were examined before and after the introduction of a multidisciplinary team. Of the 243, 117 were studied before and 126 were studied after the inception of an MDT and there were no differences in age, sex and extent of deprivation between the two cohorts [11]. Three years after the introduction of a MDT, 23% received chemotherapy, compared to only 7% in the year prior to the inception of MDT while 44% of post MDT patients received palliative care only compared to 58% of pre-MDT patients. In addition, median survival post-MDT was 6.6 months, compared to 3.2 months pre-MDT [11]. This study suggests that MDT management of cancer patients not only results in the change of treatment of patients, but also significantly increase survival. Several other studies have also suggested that MDT management improves patients’ quality of life and survival and is vital for second opinions [12, 13].

Fader and colleagues also evaluated the cost effectiveness of multidisciplinary care in Michigan, United States of America [14]. They studied the cost effectiveness of the Multidisciplinary Melanoma Clinic (MDMC) compared to the traditional community-based treatment. In the study, a consecutive sample of 104 patients with local disease who were treated in the Michigan community was compared with 104 blindly selected subjects treated at the MDMC during a similar time period. The authors concluded that multidisciplinary care would save $1600 (£969) per patient when compared with the group treated in the community.

In another study by Gabel and colleagues, they evaluated patients’ satisfaction in the management of newly diagnosed breast cancer patients before and after the establishment of a multidisciplinary breast cancer clinic (MDBCC) [15]. 166 patients who were referred in the traditional consultation manner were evaluated in the control group during the year prior to the establishment of MDBCC. They were compared with the first 177 patients seen during the first year of the clinic's operation. The authors reported that MDBCC increased patient satisfaction by encouraging input of patients′ families and friends and by helping patients make treatment decisions (P < 0.001) [15]. They also found that the time between diagnosis and the initiation of treatment was considerably reduced (42.2 days vs. 29.6 days; P < 0.0008).

Only very few publications have suggested that the multidisciplinary team management of cancer patients is ineffective. Acher et al studied 124 cases of urological cancers discussed in 10 MDT meetings over a six month period and discovered that only two cases had their clinical management changed as a result of the meeting, while four changes were made due to histological reports and 1 to radiology, none of which had an effect on clinical management [16]. Although the result of this study may not have shown a statistical significance, the fact that two patients had their clinical management changed is of clinical significance because this could have an effect on the morbidity and mortality of the patients who had their clinical management changed.

### Team's perspective

It has been shown that many MDT members believe that MDT meetings are very effective. In a study of 253 Colorectal Surgeons and Colorectal Clinical Nurse Specialists by Sharma and associates, it was revealed that 96.5% of respondents believed that MDTs improved the overall quality of care of colorectal cancer patients and 73% thought that MDTs were cost effective [17].

### Barriers to Multidisciplinary Teamwork

Although it is well known that the multidisciplinary team management of cancer patients generally improve patient outcome, there are a number of barriers that prevent the full realisation of these benefits. Such barriers include insufficient facilities, time constraint and poor interprofessional relationships. Furthermore, some studies have shown that in some cases, decisions made at MDT meetings are not implemented [18]. Blazeby and colleagues reported in their study that 41 of the 273 decisions made by the MDT were not implemented [18]. Having an MDT in small hospitals may also be particularly challenging as all the required specialists necessary for an MDT may not be available in such hospitals.

## Discussion

In general, many of the published studies suggest that multidisciplinary team meeting is valuable in the management of patients with cancer. These studies have been carried out mostly in the United States of America and the United Kingdom. It would be worthwhile if studies could also be conducted in several other countries so as to identify any differences in cultural or medical practice that might have an impact on MDT.

The methodology of many of the published studies appears flawed. The studies frequently used before-and-after designs, which are considered as weak evidence for determining causal relationships because of multiple potential confounders. New designs should be looked into in future studies and there should be an adjustment for any confounder in case-control studies. Randomised control trials may actually be the best tool to evaluate the effectiveness of the multidisciplinary team.

Multidisciplinary team meetings (MDTs) form part of the daily activities in many hospitals caring for cancer patients as a form of institutionalised communication. The degree of organisation and the type of communication in these MDTs has a direct impact on the quality of patient care provided. Undoubtedly, following the Calman-Heine report, the inception of the MDT in many hospitals has resulted in better management of cancer patients and has resulted in improved quality of life for such patients. One resulting decision from a multidisciplinary discussion is more accurate and effective than the sum of all individual opinions. Also, even when individual opinions are accurate in some cases, the MDT meetings provide assurance on the accuracy of such decisions. In other words, the MDT provides important second opinions for patients. Furthermore, through active discussion and retrospective cases review in meetings, specialists gain valuable experience of how treatments can be combined to optimise patient outcome. Multidisciplinary team management ensures that the standard of patient care is consistent, which was a major problem before the inception of MDT in many hospitals. MDT meetings also provide a valuable avenue of teaching for medical students and junior doctors, and also improve communication between different specialists. The author of this paper has himself attended a number of MDT meetings at the University Hospital of Wales, Cardiff and Princess of Wales Hospital, Bridgend, and found them a valuable learning avenue.

An MDT requires mature leadership to create a democratic climate that allows for open and constructive discussion. Controversies, which are inevitable within a team that is striving to reach decisions concerning complex situations, thus require a variety of approaches for dealing with them when they occur.

In order to tackle barriers to MDT meetings such as time constraints, the meetings should be appropriately integrated into the work schedule of specialists and be given equal weighting just as ward rounds or theatre sessions. Also, it may be helpful for small or district hospitals to have facilities for teleconferencing so that specialists do not have to travel to the large teaching hospitals to attend these meetings. Finally, the progress of patients should be reviewed regularly in consequent multidisciplinary team meetings to ensure that decisions taken previously are implemented appropriately.

With increasing emphasis on MDT management of cancer patients, future doctors in almost all specialties are more likely to be involved in multidisciplinary cancer care. Not only surgeons and medical oncologists would be involved in the management of cancer patients, but also general practitioners, pathologists, radiologists, palliative care specialists and psychiatrists. Therefore, it is essential for medical students to become familiar with the principles and practice of multidisciplinary teams early in their medical careers. This will promote interdisciplinary interactions that will facilitate effective teamwork. In fact, some studies have suggested that in the future, cancer patients may be involved in MDT meetings [19].

## Conclusion

With the increasing incidence of cancer and growing complexity of cancer care, multidisciplinary teams have the potential of improving the quality of life and increasing survival of cancer patients. Although there are a number of challenges to the success of the MDT, continuous commitment and support will ensure that MDT achieve its full potential in delivering effective and efficient cancer care. Finally, more prospective studies should be conducted to further evaluate the efficacy of the MDT and to highlight factors that may be militating against its success so that such factors could be addressed to ensure that patients receive the best possible care.
